# A Changing Model for Developing Health Products for Poverty-Related Infectious Diseases

**DOI:** 10.1371/journal.pntd.0003379

**Published:** 2015-01-08

**Authors:** Piero L. Olliaro, Annette C. Kuesel, John C. Reeder

**Affiliations:** 1 Special Programme on Research and Training in Tropical Diseases (TDR), a co-sponsored programme of UNICEF/UNDP/World Bank/WHO, and based at the World Health Organization, Geneva, Switzerland; 2 Centre for Tropical Medicine, Nuffield Department of Medicine, University of Oxford, Oxford, United Kingdom; McGill University, Canada

To achieve disease control objectives, country programmes need effective tools for prevention, diagnosis, and treatment, but the current pharmaceutical profit-driven motive neglects “tropical diseases.” Over the 40 years of its existence, TDR has used a range of approaches to promote the development and effective use of new products. Many were successful and contributed to identifying, testing, registering, and implementing tools for tropical disease, but lessons can also be learnt from those which failed or proved not sustainable.

This paper reviews examples of TDR approaches and contributions to drug discovery research and development (R&D) and the optimisation of existing treatments against the backdrop of vast changes in the R&D landscape for infectious diseases of poverty. New funders and organizations are now available to conduct product R&D, allowing TDR to reduce its own R&D role to one of facilitation and promotion of an environment more conducive to innovation in R&D and access to the resulting products. Our focus is now more on intervention and implementation research to generate the evidence needed for decisions on where, when, and how products can best be incorporated into national health services for maximum benefit to the people in need.

## Where Have Drugs for Tropical Diseases Come from during the Past 40 Years?—TDR Changing Roles

Before the 1990s, drug R&D was almost exclusively driven by the pharmaceutical industry, which had retained some interest in tropical diseases, mostly for travellers. The public sector provided support but was generally not directly involved with drug development and registration. Accordingly, in its early days TDR invested into defining research needs and into basic and discovery research (in particular the development of models for compound screening). This was expected to generate development candidates for the pharmaceutical industry to take on and further develop into products. As it would later become apparent, this was not enough, and a more active participation of the public and not-for-profit sector was needed.

Between 1975 and 1990 [1], ten drugs were registered for tropical diseases: benznidazole and nifurtimox for Chagas disease; oxamniquine and praziquantel for schistosomiasis; pentamidine for human African trypanosomiasis (HAT); pyrazinamide, halofantrine, mefloquine for malaria; albendazole for soil-transmitted nematodes (STNs); and ivermectin for onchocerciasis. It is worth noting that of these ten drugs, all but halofantrine are still in use. Praziquantel, ivermectin and albendazole were not the results of a focussed R&D effort for neglected diseases, but were originally developed as animal health products and then repurposed for human disease. Developed 25–40 years ago, these drugs are still the cornerstone of current disease control strategies. Is this impressive longevity of therapeutic/control utility, or failure of the system to generate new drugs, especially in the face of looming or emerging parasite resistance?

While collaboration for product development between private and public sector was not systematic, exceptions existed. Mefloquine was developed in collaboration between the United States Army Medical Research and Development Command (Division of Experimental Therapeutics, Walter Army Institute of Research [WRAIR]), Hoffman-La Roche and TDR. This public–private partnership provided a much-needed alternative to chloroquine and antifolates, which were already failing in areas of the world.

TDR recognised early on the need to protect drugs from resistance through drug combinations. Its advocacy for and involvement in development of combination treatments began in the late 1970s when primary and secondary resistance to dapsone developed; with financial support from the WHO Leprosy Unit, multidrug therapy for leprosy was tested in clinical trials, which revolutionized the treatment of leprosy [2]. Unfortunately, for malaria options were limited and the lifespan of the mefloquine plus sulfadoxine-pyrimethamine combination in Southeast Asia was very short. While TDR was initially slow in recognising the potential of artemisinin for malaria, it was later instrumental in developing and promoting artemisinin-based combinations (see below).

In the 1990s, it became increasingly clear that relying on the private sector would not deliver much-needed medicines for poor countries, as industry was withdrawing from tropical medicine. TDR consequently decided to play a more active role [3]. The gap in pharmaceutical investment and the success of the mefloquine development partnership provided a good rationale for TDR to form additional public–private partnerships for new product R&D for tropical diseases. A TDR product development unit was formed for drugs for malaria, kinetoplastidae, and filariae, and initially also for vaccines for malaria, leprosy, and schistosomiasis. Its mandate was to go beyond the definition of strategic needs, basic research, and drug discovery of the earlier years and to develop new products through funding of investigator-initiated or TDR-solicited projects, if possible in strategic partnerships with other public or private partners.

Trouiller et al.'s [1] report an additional six products registered during this period: eflornithine for stage 2 human African trypanosomiasis, liposomal amphotericin B for visceral leishmaniasis, injectable artemether for severe malaria and atovaquone for uncomplicated malaria, and rifabutin and rifapentine for tuberculosis. TDR had an active role in the development of eflornithine, liposomal amphotericin B, and artemether and provided the coordination and funding of the pivotal clinical trials that supported their registration.

Through programmes started in the 1990s, TDR played a pivotal role in the development of six of the 22 products for neglected diseases (12 for malaria, three for leishmaniasis, seven for tuberculosis) registered or WHO-prequalified during 2000–2011 [4]. TDR led or participated actively in the development of three (injectable artemotil [arteether] and rectal artesunate for malaria; and oral miltefosine for visceral leishmaniasis [VL]), and was a contributor to the identification and initiation of a number of others (artesunate-amodiaquine and artesunate-mefloquine for malaria; paromomycin for VL).

These contributions used early models of the public-private product development partnerships later applied more broadly to the development of products for poverty-related infectious diseases. These partnerships “de-risk” drug development through financial and “in-kind” contributions to R&D by public and not-for-profit organisations, and the resulting de-linking of costs of development and pricing forms the basis for negotiating preferential pricing and donations.

Concurrently, TDR continued to fund research into new targets, compound screening, and identification of leads and development candidates. This involved setting up and supporting centres for compound screening as well as networking researchers and institutions. Agreements with pharmaceutical companies allowed the screening centres to access company compound libraries in addition to those from academic groups. Importantly, TDR invested into strengthening capacities in both discovery and clinical research in disease-endemic countries to empower the countries and make these efforts sustainable locally in the long-term. This large and increasingly coordinated R&D investment produced both failures and significant results, with compound series and leads later passed on to other organisations or made publicly available.

Towards the end of the 1990s it became clearer that TDR was not ideally suited to perform and coordinate the number of diverse activities that development requires, and that it could not sustain such efforts. TDR lacked the necessary narrow product focus and the flexibility in fundraising and engaging with the private sector. Alternative solutions were sought, and TDR independent product development partnerships (PDP) seemed the logical way forward. This led TDR initially to support the creation of the first-ever PDP for tropical disease R&D, the Medicines for Malaria Venture (MMV), followed by a prominent role in the establishment of the Drugs for Neglected Diseases initiative (DNDi), the incubation of the Foundation for Innovative New Diagnostics (FIND), and contribution to the initial phases of the Global Alliance for tuberculosis drug development (GATB). TDR's experiences with its own public–private partnerships provided valuable insights for these new ventures.

With PDPs coming of age in the 2000s, TDR started to shift its focus again, transferring R&D projects to other organisations to the extent possible and increasing funding for post-R&D intervention and implementation research to provide the evidence for WHO recommendations and ministries of health policy decisions on where, when, and how interventions should be integrated into the health systems and communities.

Some of the approaches, products, and interventions developed over 40 years have had major public health impacts, and also shaped the research and disease-control agenda for other organizations. For instance, there was research which provided the basis for the strategy of the African Programme for Onchocerciasis Control: rapid epidemiological mapping of onchocerciasis and community-directed treatment with ivermectin [5]–[7]. Another example is evidence that supported a paradigm change in the treatment of malaria from single-agent to artemisinin-based combination therapy (ACT). This project, led by TDR and the Wellcome Trust, started in the late 90s and benefitted from early dialogue and involvement of malaria control programmes, an essential element in facilitating policy uptake. Other projects turned out to be less relevant or unsuccessful. Of course, a proportion of compounds will inevitably fail in the R&D process, but in some cases the choice of the product or interventions or the timing was not ideal (e.g., injectable arteether is hardly ever used for severe malaria), the collaboration was not successful, or the final pricing restricted accessibility of the product.

## The Environment Keeps on Changing; How Is TDR Responding?

In the 40 years of TDR's existence, the goal posts have shifted. Though infectious diseases continue to cause significant morbidity and mortality, disproportionately affecting the poorest members of societies, they no longer affect primarily low-income countries. The bulk now lies in middle-income countries, and inequalities in living conditions and access to health care are increasing in all geographic areas. This is occurring at a time when the pharmaceutical industry is turning away from infectious diseases, leaving many health issues unattended.

Many of the products that we need to control these diseases are simply not there yet: the next generation of antimalarials, shortened tuberculosis treatments, and alternatives for one-drug programmes, for example, or suitable diagnostics and tests of cure. A main focus of TDR's new strategy is therefore to foster innovation in R&D and improve access to healthcare treatments. The traditional mechanism of funding and conducting R&D is probably reaching its structural limits and may not be sustainable in the long-term. More innovative solutions are required to overcome obstacles such as the narrow funding base of R&D and intellectual property barriers. Better coordination between players is also essential to prevent duplication and ensure efficient progress. We believe that processes can be optimised and time and money saved by more open sharing of information and knowledge and more proactive involvement of the countries primarily concerned. Emerging economies already have capacities for developing new anti-infectives, and organizations such as the African Network for Drugs and Diagnostic Innovation (ANDI), incubated by TDR but now independent, are becoming a strong voice for the involvement of disease-endemic countries.

While the search for new products continues, there is also the challenge of having what is already available effectively deployed and used. One focus of TDR's new strategy is, therefore, research to translate “products” into interventions and strategies that work at scale. By combining older or newer medicines with preventative measures such as vector control and other effective interventions, it is now possible to target enhanced control and even elimination of diseases like visceral leishmaniasis, schistosomiasis, lymphatic filariasis, onchocerciasis, and malaria, at least in some regions. The challenge is to generate the evidence base for the effective delivery of combined interventions. TDR's decision to concentrate on more “downstream” questions is addressing these practical problems. We now work with concerned countries to test first and then validate and apply at scale combined interventions to achieve those ambitious objectives. An example is the research conducted jointly with the research and control communities of Bangladesh, India, and Nepal that provides evidence for interventions deployed in the visceral leishmaniasis elimination programme in the Indian subcontinent [8].

## Looking Backwards and Forward

When TDR was formed, it was essentially the only “game in town.” The early work provided critical foundations in a number of areas and led to important new treatments. TDR experimented with many approaches over the years, some of which worked and some of which did not. Those that worked did not only have an impact on public health but also planted seeds for further innovation and progress. Some of the approaches that didn′t work contributed to modifications in TDR's way of working.

Today's challenges are different than those at TDR's start. There are different players and more funding, still primarily public and/or not-for-profit, devoted to R&D for new drugs and diagnostics, requiring coordination for optimization of resources. Getting the new drugs to those who need them is as big a challenge as developing them. This dynamic environment requires innovative solutions, which need both continuity and adaptation in order to produce the intended results.

Today, TDR is rarely directly involved in drug R&D but instead works with individual organizations, partnerships, and countries to foster innovation and R&D for new products needed, as well as to help identify and implement long-term solutions.
